# Precision oncology for NSCLC: a bibliometric exploration of emerging trends and research priorities

**DOI:** 10.1097/JS9.0000000000003177

**Published:** 2025-08-11

**Authors:** Liping Zhang, Yanhui Yang, Tianhu Wang

**Affiliations:** a Department of Thoracic Surgery, Third Affiliated Hospital of Chongqing Medical University, Chongqing, China; b Department of Thoracic Surgery, The First People’s Hospital of Neijiang, Neijiang, China

*Dear Editor,*

Non-small cell lung cancer (NSCLC) accounts for approximately 85% of all lung cancer cases and remains the leading cause of cancer-related deaths worldwide, with an estimated 1.8 million deaths annually[1]. Recently, precision therapy has shown promise in improving outcomes by tailoring treatment based on individual genetic and molecular profiles. This approach, which includes the use of tyrosine kinase inhibitors, immune checkpoint inhibitors, and next-generation sequencing, is reshaping the clinical management of NSCLC, offering more effective and less toxic treatments^[2,3]^. As research in this field rapidly advances, there is an urgent need for comprehensive bibliometric analyses to map emerging trends, identify research hotspots, and anticipate future developments^[4,5]^. Importantly, our bibliometric analyses performed in line with the TITAN 2025 guideline[6] help ensure transparency and reliability in mapping emerging trends and research priorities.

We retrieved publications on precision therapy for NSCLC from the Web of Science Core Collection (WoSCC) on 1 April 2025, using a comprehensive search strategy for precision medicine and NSCLC. Eligible studies included English-language articles and reviews; conference abstracts and non-relevant reports were excluded. Bibliometric analyses were performed with GraphPad Prism for publication trends and CiteSpace and VOSviewer to map co-citation networks and research hotspots.

As of 31 December 2024, we identified 982 publications on NSCLC precision therapy in WoSCC, including 619 original articles and 363 reviews (Supplemental Digital Content Figure S1, available at: http://links.lww.com/JS9/E842). Research output grew markedly after 2009 and peaked in 2024 (Fig. 1A). Geographically, the United States led with 32.18% of publications, followed by China and Italy (Supplemental Digital Content Table S1, available at, http://links.lww.com/JS9/E843 (Fig 1B, C). The U.S. also had the highest citation count (11 942) and citation-to-publication ratio (37.79), reflecting its global influence. Collaboration networks show strong ties between the U.S. and China, and regional partnerships within Europe and Asia (Fig. 1D). Institutional analysis revealed 1919 contributing organizations, with Harvard University, Harvard Medical Affiliates, and the University of Texas System leading output (Supplemental Digital Content Table S2, available at: http://links.lww.com/JS9/E843). However, most collaborations occurred within countries, highlighting a need for stronger international partnerships (Fig. 1E). Cancers was the most prolific journal, while Journal of Clinical Oncology was the most co-cited, alongside Clinical Cancer Research and New England Journal of Medicine (Supplemental Digital Content Tables S3-S4, available at: http://links.lww.com/JS9/E843 (Fig 1F, G). Dual-map overlay analysis (Fig. 1H) emphasized the interdisciplinary nature of precision therapy research, spanning molecular biology, immunology, and clinical medicine. Among authors contributing to NSCLC precision therapy, the top 10 published 87 papers (8.76% of total), led by Umberto Malapelle (13 papers), Silvia Novello (11 papers), and Paul Hofman (10 papers) (Supplemental Digital Content Table S5, available at, http://links.lww.com/JS9/E843). Co-citation analysis identified Shaw AT (215 citations), TS Mok (173), and Rosell R (169) as the most influential authors, with their central roles visualized in the collaboration network (Fig. 1I).

**Figure 1.**
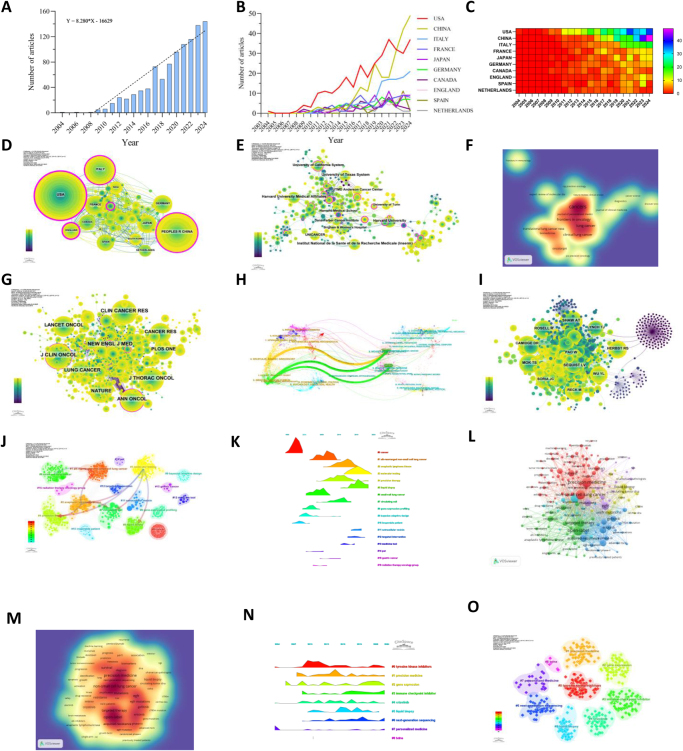
Overview of bibliometric landscape in precision oncology for non-small cell lung cancer. (A) Annual volume of publications; (B-D) Country/region collaboration network of research on Precision Oncology for non-small cell lung cancer: (B) Line graph of national publications; (C) Heat map of national publications; (D) Networks of country cooperation, node size represents the volume of publications and the line color represents the year of collaboration; (E) Networks of institutional co-operation; (F-G) Analysis of journal sources: (F) Density web map of journal publications; (G) Co-citation network map of journals; (H) Dual map of journals. (I) author. (J-K) Analysis cited references and co-cited references: (J) Clustering of co-cited literature. (K) Peak map of co-cited literature. (L-O) Analysis of keywords associated with Precision Oncology in non-small cell lung cancer: (L) Network map of high-frequency keywords; (M) Density map of keywords; (N) Peak map of keyword clustering; (O) Clustering map of keywords.

Citation and co-citation clustering revealed pivotal studies, with NEJM publications on osimertinib and alectinib ranking first and second, respectively (Supplemental Digital Content Table S6, available at: http://links.lww.com/JS9/E843). Early hotspots included “cancer” and “inoperable patient,” while recent trends focus on “ALK rearrangements,” “liquid biopsy,” and “precision therapy” (Fig. 1J, K). The strongest burst references highlight shifting priorities towards molecular diagnostics and targeted treatments (Supplemental Digital Content Figure S2, available at, http://links.lww.com/JS9/E842). Keyword analysis showed “precision medicine,” “NSCLC,” and “EGFR” as dominant terms (Supplemental Digital Content Table S7, available at, http://links.lww.com/JS9/E843, Fig 1L, M). Six major clusters were identified, with current hotspots in tyrosine kinase inhibitors, immune checkpoint inhibitors, and next-generation sequencing. Volcano plots (Fig 1N, O) and citation burst maps (Supplemental Digital Content Figure S3, available at: http://links.lww.com/JS9/E842) further emphasize these emerging themes.

To our knowledge, this is the first comprehensive bibliometric study focusing specifically on precision oncology in NSCLC, integrating recent developments such as liquid biopsy, next-generation sequencing, and immune checkpoint inhibitors. First, it provides a comprehensive temporal overview (2000–2024), capturing the evolution of research priorities from traditional targeted therapies to non-invasive diagnostics and immunotherapies. Second, using dual-map overlays and citation burst analysis, we reveal interdisciplinary linkages and shifts in collaboration patterns that were not detailed in prior analyses. Third, our identification of research gaps, particularly the underrepresentation of rare molecular subtypes, underscores the need for broader molecular profiling and global equity in precision oncology. Finally, the study highlights actionable learning points: the importance of strengthening international collaborations, improving molecular testing accessibility, and focusing future research on rare driver mutations to maximize the potential of precision medicine for NSCLC. These findings provide a roadmap for future research directions and actionable insights for policy and clinical practice.

In conclusion, this bibliometric analysis highlights the rapid growth and evolving complexity of research on precision therapy for NSCLC. The United States leads in publication output and citation impact, while China shows substantial publication growth but needs to enhance the quality and global influence of its research. Despite significant advancements, challenges remain, including the need for further international collaboration and the expansion of research on rare molecular subtypes. The analysis also reveals that precision therapies, such as tyrosine kinase inhibitors and immune checkpoint inhibitors, are revolutionizing NSCLC treatment, but the clinical adoption of non-invasive methods like liquid biopsy is hindered by sensitivity and cost challenges. Furthermore, gaps exist in the literature, with a focus on well-characterized mutations like EGFR and ALK, while rarer genetic alterations require further exploration. Moving forward, the field must address these limitations by prioritizing large-scale trials, improving accessibility to molecular testing, and ensuring that advancements in precision therapy reach diverse patient populations across regions and socioeconomic groups. This will be crucial for maximizing the potential of precision medicine and ultimately improving patient outcomes globally^[7,8]^.

## Data Availability

Full data are available upon request to the corresponding author.

## References

[1] Molina JR Yang P Cassivi SD Schild SE Adjei AA Non-small cell lung cancer: epidemiology, risk factors, treatment, and survivorship. Mayo Clin Proc 2008;83:584–94. 18452692 10.4065/83.5.584 PMC2718421

[2] Guo H Zhang J Qin C. Biomarker-targeted therapies in non-small cell lung cancer: current status and perspectives. Cells 2022;11:3200. 36291069 10.3390/cells11203200 PMC9600447

[3] Restrepo JC Dueñas D Corredor Z Liscano Y. Advances in genomic data and biomarkers: revolutionizing NSCLC diagnosis and treatment. Cancers 2023;15:3474. 37444584 10.3390/cancers15133474 PMC10340640

[4] Wang FY Yeh YC Lin SY. Real-world application of targeted next-generation sequencing for identifying molecular variants in Asian non-small-cell lung cancer. BMC Cancer 2025;25:715. 40247220 10.1186/s12885-025-14016-z PMC12004552

[5] Mitchell CL Zhang AL Bruno DS Almeida FA. NSCLC in the era of targeted and immunotherapy: what every pulmonologist must know. Diagnostics (Basel) 2023;13:1117. 36980426 10.3390/diagnostics13061117 PMC10047174

[6] Riaz AA Ginimol M Rasha R. Transparency in the reporting of Artificial Intelligence – the TITAN guideline. Prem J Sci 2025;10:100082.

[7] Soria JC Ohe Y Vansteenkiste J. Osimertinib in untreated EGFR-mutated advanced non-small-cell lung cancer. New Engl J Med 2018;378:113–25. 29151359 10.1056/NEJMoa1713137

[8] Claus J De Smet D Breyne J. Patient-centric thresholding of Cobas® EGFR mutation test v2 for surveillance of EGFR-mutated metastatic non-small cell lung cancer. Sci Rep 2024;14:18191. 39107402 10.1038/s41598-024-68350-6 PMC11303541

